# Federated Learning Optimization Algorithm for Automatic Weight Optimal

**DOI:** 10.1155/2022/8342638

**Published:** 2022-11-09

**Authors:** Xi Yu, Li Li, Xin He, Shengbo Chen, Lei Jiang

**Affiliations:** ^1^School of Computer and Information Engineering, Henan University, Kaifeng 475001, China; ^2^School of Software, Henan University, Kaifeng 475001, China

## Abstract

Federated learning (FL), a distributed machine-learning framework, is poised to effectively protect data privacy and security, and it also has been widely applied in variety of fields in recent years. However, the system heterogeneity and statistical heterogeneity of FL pose serious obstacles to the global model's quality. This study investigates server and client resource allocation in the context of FL system resource efficiency and offers the FedAwo optimization algorithm. This approach combines adaptive learning with federated learning, and makes full use of the computing resources of the server to calculate the optimal weight value corresponding to each client. This approach aggregated the global model according to the optimal weight value, which significantly minimizes the detrimental effects of statistical and system heterogeneity. In the process of traditional FL, we found that a large number of client trainings converge earlier than the specified epoch. However, according to the provisions of traditional FL, the client still needs to be trained for the specified epoch, which leads to the meaningless of a large number of calculations in the client. To further lower the training cost, the augmentation FedAwo ^*∗*^ algorithm is proposed. The FedAwo ^*∗*^ algorithm takes into account the heterogeneity of clients and sets the criteria for local convergence. When the local model of the client reaches the criteria, it will be returned to the server immediately. In this way, the epoch of the client can dynamically be modified adaptively. A large number of experiments based on MNIST and Fashion-MNIST public datasets reveal that the global model converges faster and has higher accuracy in FedAwo and FedAwo ^*∗*^ algorithms than FedAvg, FedProx, and FedAdp baseline algorithms.

## 1. Introduction

Federated learning, a distributed machine-learning framework that can effectively protect the privacy and security of user data, has received extensive attention from academia and industry in recent years. Federated learning involves co-training a machine-learning model by servers and clients. The server sends the global model to clients, receives local models trained by clients, and aggregates them to generate a new global model until the training of the global model ends. Clients use local data to train the global model given by the server and return the trained local model to the server [1]. Federated learning effectively protects the privacy and security of data by transmitting model parameters between the server and the client (data do not leave the client) and is used in many fields. The most typical example is Google's keyboard input method, which uses a federated learning platform to train a recurrent neural network (RNN) for next word prediction. In addition, federated learning is also widely used in clinical auxiliary diagnosis, new drug development, and precision medicine in the medical industry, portrait recognition, and voice print recognition in the security industry. Although federated learning effectively solves the problem of data privacy and security, it is different from traditional distributed machine learning and brings serious challenges to system heterogeneity and statistical heterogeneity. Traditional distributed machine learning is usually deployed in the same data center or in a network with a good communication environment, and the clients for model training have similar hardware conditions. However, the clients of federated learning are often widely distributed in geographical locations. There are great differences among them in network conditions, hardware environment, and computing power, and the time when clients can participate in model training is also different. The above phenomenon is called system heterogeneity which may lead to the problems of falling behind (nodes that cannot complete the specified training rounds within the specified time) and fault tolerance [2]. In addition, data distribution and data volume of local data held by different clients are also different, which is the statistical heterogeneity of data. Both statistical heterogeneity and system heterogeneity have a negative impact on the convergence speed and final accuracy of the global model [3].

At present, most researchers try to reduce the negative impact of heterogeneity by sampling clients and modifying clients' loss function. The sampling method is that the server filters out the local models that are more conducive to the global model convergence for aggregation. In sampling algorithms [4, 5], the method of importance is widely used [4, 6–8]. This method selects the “important” clients by comparing client gradient information and aggregates their local gradients. The method of modifying the loss function of the client is more mainstream at present [2, 9, 10]. Its idea is to modify the loss function of the client, such as adding a near term [2] in the loss function or normalizing it with the last round of the global model [11, 12]. However, the above methods ignore a crucial phenomenon: the imbalance of computing power between servers and clients in the federated learning system. We know that in the actual application scenario, the computing power of clients is relatively weak. The method of modifying the client loss function further increases the computing burden of the client. Servers often have strong computing power and network conditions, and they only undertake the task of aggregating local models and generating global models.

Obviously, in the federated learning system, the computing power and network environment of clients are poor, but they are responsible for heavy work of model training. The server with the strong computing power and network environment undertakes light work, which does not match its ability. In order to make better use of system resources and improve performance, this paper studies how to use server resources to solve the problems of statistical heterogeneity and system heterogeneity without increasing the load of clients. This paper proposes the federated learning algorithm for automatic weight optimization (FedAwo) and its enhancement algorithm (FedAwo ^*∗*^) and verifies the feasibility of the methods from both theoretical and experimental aspects. Our main contributions in this paper are as follows:

1. We design a federated learning algorithm for automatic weight optimization (FedAwo). In this algorithm, the server calculates the optimal weight for the local model through the machine-learning algorithm to solve the problem of statistical heterogeneity and system heterogeneity in federated learning. The FedAwo algorithm effectively utilizes server resources and does not increase the burden on clients.
2. We prove the convergence of FedAwo and propose the enhancement algorithm FedAwo ^*∗*^ for FedAwo to further reduce the training cost. The algorithm of FedAwo is based on the heterogeneity of clients, and FedAwo ^*∗*^ reduces the training cost by dynamically adjusting the training epoch times of local model training.
3. We use the MNIST and Fashion-MNIST public datasets as test datasets and use FedAvg and FedProx as baseline algorithms to compare the performance of them with that of FedAwo and FedAwo ^*∗*^ under IID and non-IID conditions. The analysis results show that the FedAwo and FedAwo ^*∗*^ algorithms can converge faster and obtain a better global model. The experimental code of this article has been uploaded to Github (https://github.com/amazing.yx/FedAwo).

The rest of this paper is organized as follows: The second section introduces the related work of federated learning in solving heterogeneity. The third section introduces the federated learning algorithm for automatic weight optimization (FedAwo) in detail. In the fourth section, we prove the convergence of the FedAwo algorithm. In the fifth section, we propose the optimization algorithm FedAwo ^*∗*^. In the sixth section, we verify the performance of FedAwo and FedAwo ^*∗*^ through experiments. Finally, we summarize this paper.

### 1.1. Related Work

The research studies on the convergence of federated learning [2, 9, 11, 13] show that the system heterogeneity and statistical heterogeneity in federated learning have a great negative impact on the convergence speed and accuracy of the global model.

The optimization methods of heterogeneous problems mainly focus on modifying the loss function of clients or sampling clients. For modifying the loss function of the client, literature [2] proposed the FedProx algorithm, which aims to add a proximal term (*μ*/2)‖*x* − *x*^(*t*, 0)^‖^2^ to help improve the stability of federated learning. At the same time, the FedProx algorithm would dynamically adjust the number of client-training epochs to solve the straggler problem caused by system heterogeneity. The effect of this method is more obvious in the environment with stronger heterogeneity. However, the original intention of the FedProx algorithm is to solve the problem of straggler. Due to the introduction of the proximal term, the computing overhead of the client increases instead. In some cases, the problem of client struggling is even more serious. Literature [11] proposes the SCAFFOLD algorithm, which corrected the client-drift phenomenon that occurs in the FedAvg algorithm by introducing the correction term (*c* − *c*_*i*_). Literature [10] proposed the FedNova algorithm, which eliminated objective inconsistencies and maintained fast convergence by normalizing local models. The SCAFFOLD algorithm and the FedNova algorithm are the same as the FedProx algorithm. Although the communication overhead has been further optimized and the model quality has been improved, it still increases the computing overhead of the client. Literature [14] proposed the FedDyn algorithm to keep the local model and global model distribution approximately consistent by assigning a dynamic regularization optimizer to each client in each round. All of these methods can reduce the influence of heterogeneity on the convergence speed and model accuracy, but they all increase the computational overhead of clients. The computing power of the server is better than that of the client. In practice, most clients are always busy, but the server is often idle.

For the sampling method, the authors in [4] established a general sampling-federated learning system and obtained an unbiased optimal sampling probability to alleviate the influence of heterogeneity on the global model. Literature [15] proposed the FedL algorithm, which was a graph convolutional network (GCN)-based sampling method that maximized the accuracy of the global model by learning the relationship between network attributes, sampling nodes, and generated offloads. Literature [16] classified local models according to the importance^ of each round of clients, aggregated the “important” local models, and proposed an approximate unbiased sampling optimization algorithm. Literature [17] proposed the FOLB algorithm by estimating the gradient information of the local model, which inferred the performance of the client and performed weighted sampling based on it. This method could cope with system heterogeneity and made the global model converge quickly. Although the sampling method can promote the global model to converge quickly, the quality of the final global model is poor.

In addition, literature [18] proposed the FedHQ algorithm to solve the system heterogeneity by minimizing the upper limit of the convergence speed as a function of the heterogeneous quantization error of all clients and assigning different aggregation weights to different clients. In order to address heterogeneity, literature [19] proposed an algorithm with periodic compressed communication, which introduced a local gradient tracking scheme and obtained fast convergence speed matching communication complexity. Literature [20] analyzed the convergence bound of gradient descent-based federated learning from a theoretical perspective and obtained a novel convergence bound. Using the above theoretical convergence bound, literature [20] proposed a control algorithm that learns data distribution, system dynamics, and model characteristics, and based on which, it dynamically adapts the frequency of global aggregation in real time to minimize the learning loss under a fixed resource budget. Literature [18–20] solved the system heterogeneity caused by external environment such as system configuration and hardware conditions, but do not pay attention to the statistical heterogeneity caused by local data differences.

Due to the limitations of the above two methods, this paper hopes to solve the problem of heterogeneity by introducing adaptive learning. Before that, literature [13, 21, 22] tried to combine adaptive learning with federated learning. Literature [21] proposed a federated learning optimization scheme with an adaptive gradient descent function. This algorithm improved the privacy performance of the local training process by differential privacy and the scaling of update volume. This algorithm can enhance the privacy security of each client in the process of joint learning, but it cannot effectively suppress the negative impact of heterogeneity. Literature [22] proposed an adaptive-personalized federated learning (APFL) algorithm, where each client would train their local models while contributing to the global model. The APFL algorithm adaptively learns the model by leveraging relatedness between local and global models as learning proceeds, which effectively improves the convergence speed of the global model. Literature [13] proposed federated adaptive weighting (FedAdp) that assigns different weights to nodes for global model aggregation in each round of communication. The FedAdp algorithm allocates the weight of the client by calculating the intercept between the global model and the local model. However, when the performance of the local model is due to the global model, FedAdp will still assign a lower weight to the local model according to the intercept value, which is obviously unreasonable. We summarize the limitations of the above methods in Table 1.

Therefore, the method of modifying the client loss function increases the computational overhead of the client, and the sampling method has the problem of low accuracy of the final global model. However, the current federated learning algorithm combined with adaptive learning does not focus on solving the problem of heterogeneity. This paper is different from the above methods. From the perspective of resource allocation of the federated learning system, this paper makes full use of the advantageous resources of servers and combines adaptive learning to reduce the negative impact of heterogeneity. As far as we know, this paper is the first work aimed at using server-computing resources to solve the optimal weight allocation value.

## 2. Federated Learning Algorithm for Automatic Weight Optimization (FedAwo)

In this section, we establish the system architecture and propose the automatic weight optimization algorithm FedAwo. Finally, we introduce the specific process of it in detail.

### 2.1. System Model

A federated learning system generally includes one server and *K* clients. The server plays the role of coordinating the training for each client, aggregating, and distributing the global model. Clients hold their own local dataset {*D*_1_, *D*_2_,…, *D*_*K*_}, and the total amount of data of all clients is ∑_*k*=1_^*K*^|*D*_*k*_| [23–27]. Clients perform a local learning operation under the coordination of servers. We first define *f*(*θ*) as a loss function, where *θ* is the model parameter. Thus, the global loss function of clients can be defined as

(1) $$\begin{array}{c} \min_{\theta \in R^{d}}\;\left\{f\left(\theta \right)\overset{de\;f}{=}\overset{K}{\underset{k=1}{\sum }}p_{k}\cdot f_{k}\left(\theta \right)\right\}\text{.} \end{array}$$

The local loss function for each client is defined as

(2) $$\begin{array}{c} f_{k}\left(\theta \right)\overset{de\;f}{=}\frac{1}{\left|D_{k}\right|}\sum_{x\in D_{k}}l_{k}\left(\theta ,x\right)\text{,} \end{array}$$

where *l*_*k*_(*θ*, *x*) is the loss function evaluated at the data sample *x*, and the model *θ*.*p*_*k*_ represents the training data weight value of the *k*-th client

(3) $$\begin{array}{c} p_{k}=\frac{\left|D_{k}\right|}{\overset{K}{\underset{k=1}{\sum }}\left|D_{k}\right|}\text{.} \end{array}$$

The global model aggregation mode is defined as

(4) $$\begin{array}{c} \theta^{t+1}=\overset{K}{\underset{k=1}{\sum }}p_{k}\cdot \theta_{k}^{t}\text{.} \end{array}$$

The purpose of federated learning is to find the optimal value in (1), and the FedAvg algorithm is to repeat the process of (3) and (4) until the global model converges. The most popular and de facto optimization algorithm to solve (1) is FedAvg [1]. Here, denoting *t* as the index of a federated learning round, we describe one round (e.g., *t*-th) of the FedAvg algorithm as follows:

1. The server uniformly broadcasts the global model *θ*^*t*^ to each client.
2. Each client uses local data to perform local SGD to calculate the updated model *θ*_*k*_^*t*^. Then, the client sends the updated model back to the server.
3. The server aggregates (with a weight *p*_*k*_ ) the clients' updated model and computes a new global model *θ*^*t*+1^.

The above process repeats for many rounds until the global loss converges.

At present, the research on the negative effects of heterogeneity mostly uses the sampling method or modifies the loss function of clients. Different from the previous algorithms, we modify *p*_*k*_ in (1) to reduce the influence of heterogeneity on the global model by finding the correction value *q*_*k*_. So the global model aggregation mode is rewritten as

(5) $$\begin{array}{c} \theta^{t+1}=\overset{K}{\underset{k=1}{\sum }}q_{k}\cdot \theta_{k}^{t}\text{.} \end{array}$$

As shown in Table 2, the loss function of the global model is updated to *θ*^*t*+1^=∑_*k*=1_^*K*^*q*_*k*_ · *θ*_*k*_^*t*^.

#### 2.1.1. Federated Learning Algorithm for Automatic Weight Optimization

We design a federated learning algorithm FedAwo for automatic weight optimization to obtain *q*_*k*_. The FedAwo algorithm aims to reduce the negative impact of statistical heterogeneity and system heterogeneity on federated learning and makes full use of the computing resources of the server. Compared with traditional federated learning, this algorithm needs to have a certain amount of high-quality data in the server, which is achievable in most federated learning tasks. We would use these high-quality data as the server's datasets in the server and use the way of machine learning to calculate the optimal weight correction value *q*_*k*_^ ^*∗*^^. The specific process of the federated learning algorithm for automatic weight optimization is as follows:

(1) The server *S* establishes a federated learning global model *θ*^*t*^ and a weight allocation model *ϑ*^*t*^. Then, the server *S* calculates the initial weight value *q*_*k*_^0^ for each client according to the data quantity. The initialization weight distribution formula of each client is *q*_*k*_^0^=(|*D*_*k*_|/∑_*k*=1_^*K*^|*D*_*k*_|), and according to the above formula, we can get the initial client weight allocation vector *γ*=[*q*_1_, *q*_2_,…, *q*_*K*_]. At the same time, the global model *θ*^*t*^ is broadcast to each client *k*, and the server has the dataset *D*_*s*_. The data in *D*_*s*_ are independent and identically distributed(IID) high-quality data. The total amount of data are *J*, and each data has a unique corresponding label *L*_*j*_, which is a one-hot type data. For example, in the MNIST dataset, the one-hot type label of digital zero is [1,0,…, 0]. We can get a matrix of all data labels *ω*=[*L*_1_, *L*_2_,…,*L*_*J*_]^T^.
(2) Each client would use its own local data for SGD for the received global model *θ*^*t*^ until it is trained for the specified criterion, and send the model *θ*_*k*_^*t*^ to the server *S*.
(3) Assuming that *D*_*s*,*j*_ is a data sample in the dataset *D*_*s*_, we input data *D*_*s*,*j*_ into the local model *θ*_*k*_^*t*^, and the output is *M*_*k*_^*j*^ , which is a one-hot type data. Then, we input all the data in *D*_*s*_ to get a matrix *M*_*k*_=[*M*_*k*_^1^, *M*_*k*_^2^,…,*M*_*k*_^*J*^]^T^. We carry out the above operations on all client models to get a matrix *M*=[*M*_1_, *M*_2_,…, *M*_*K*_]
(4) The server calculates *χ*, which is the product of *M* and *γ*. Thus, we have
  (6) $$\begin{array}{c} \chi =M\cdot \gamma =\left[\begin{array}{c} q_{1}M_{1}^{1}+q_{2}M_{2}^{1}+\cdots +q_{K}M_{K}^{1}\\ \cdots \cdots \\ q_{1}M_{1}^{j}+q_{2}M_{2}^{j}+\cdots +q_{K}M_{K}^{j}\\ \cdots \cdots \\ q_{1}M_{1}^{J}+q_{2}M_{1}^{J}+\cdots +q_{K}M_{K}^{J} \end{array}\right]\text{.} \end{array}$$
Note that each element *q*_1_*M*_1_^*j*^+*q*_2_*M*_2_^*j*^+⋯+*q*_*K*_*M*_*K*_^*j*^ in *χ* represents the average prediction result for the *j*-th sample in *D*_*s*_. We then calculate the cross-entropy loss between *χ* and *ω*, i.e.,
  (7) $$\begin{array}{c} H\left(\chi ,\omega \right)=-\overset{J}{\underset{j=1}{\sum }}\left(\chi \cdot \log\left(\omega \right)+\left(1-\chi \right)\cdot \log\;\left(1-\omega \right)\right)\text{,} \end{array}$$
where *H*(*χ*, *ω*) reflects the prediction loss under the current weight *γ*. By minimizing *H*(*χ*, *ω*), we can obtain the best weight *γ*^*∗*^, which is given by
  (8) $$\begin{array}{c} \gamma^{*}={\left[q_{1}{\;}^{*},q_{2}{\;}^{*},\cdots ,q_{k}{\;}^{*}\right]}^{T}=\underset{\gamma }{argmin}\left\{H\left(M\cdot \gamma ,\omega \right)\right\}\text{.} \end{array}$$
We take *q*_1_ ^*∗*^, *q*_2_ ^*∗*^, ⋯, *q*_*k*_ ^*∗*^ in *γ*^*∗*^ as the optimal weights. In this paper, we adopt a machine-learning-based approach in the server to get *γ*^*∗*^. In particular, a neural network model *ϑ*^*t*^ is trained so that *H*(*M* · *γ*, *ω*) is minimized.
(5) The server *S* aggregates the models according to the current round of updated weight correction values *q*_*k*_^*∗*^ to obtain the global model of the next round *θ*^*t*+1^=(∑_*k*=1_^*K*^*q*_*k*_)^*∗*^ · *θ*_*k*_^*t*^.
(6) The server broadcasts the new global model *θ*^*t*+1^ to each client and repeats the process of 1–6 until the global model *θ*^*T*^ converges.

For Algorithm 1, we need to define the initial global model *θ*^0^, the initial adaptive learning model *ϑ*^0^, and the initial weight value *q*_*k*_^0^. The server broadcasts the global model *θ*^0^ to all clients within the specified time *T* of the system. The client uses local data to train the model to the specified epoch *I* and then returns the model *θ*_*k*_^*t*,*I*^ to the server. This process is shown in 2 − 5 of Algorithm 1, which represents the process of local model training. Then, in the server, the optimal weight value *q*_*k*_^*∗*^ is obtained through the adaptive learning model *ϑ*^0^. The model aggregation is carried out according to the optimal weight value *q*_*k*_^*∗*^, and the latest global model *θ*^*t*+1^ is obtained. This process is shown in 6–9 of Algorithm 1, which represents the process of model aggregation [28–33].

The federated learning algorithm of automatic weight optimization adds an adaptive weight allocation algorithm to the FedAvg algorithm. In the traditional weight allocation method (3), the weight of the client is allocated according to the amount of data, which is fully applicable under the condition of IID. However, under the influence of heterogeneity, only considering the amount of data cannot fully reflect the quality of client data because the data of most clients tend to shift to a certain feature in practice affected by statistical heterogeneity. In other words, most data in one client often have similar features. If such a client has more data, it would often lead to a poor aggregation effect according to (3). The correct approach is to adjust the weight to minimize the cross-entropy. When the cross-entropy is the smallest, predicted local distribution is closest to global distribution, which is also the biggest advantage of FedAwo compared with the traditional weight allocation algorithm. FedAwo can converge quickly and improve the accuracy of the global model, which is still applicable under IID conditions.

## 3. Proof of Convergence

### 3.1. Nonconvex Loss Functions

As is known to all that for convergence of nonconvex loss functions, the expected gradient norm is usually taken as the index of convergence to ensure convergence to a stagnation point [15–17, 34]. Therefore, this article takes the norm of the expected gradient as the convergence index, namely,

(9) $$\begin{array}{c} \frac{1}{T}\overset{T}{\underset{t=1}{\sum }}E{\left\|\nabla f\left(\theta_{t}\right)\right\|}^{2}\leq \varpi \text{.} \end{array}$$

As is commonly used in literature studies [20–22], the following assumptions are adopted in this article.

**Assumption 1 .**

The loss function *f*(), .*f*_*k*_(), .*l*(.), *areallL*− smooth, for any *α*, *β* ∈ *R*^*d*^ and any *x* ∈ *D*, there is inequality (10).

(10) $$\begin{array}{c} \left\|\nabla l\left(\alpha ,x\right)-\nabla l\left(\beta ,x\right)\right\|\leq L\left\|\alpha -\beta \right\|\text{,} \end{array}$$

where *L* denotes the Lipschitz constant.

**Assumption 2 .**

Stochastic gradients in clients are unbiased, and the second raw moment of a stochastic gradient for all functions is *f*_*k*_. (bounded).

(11) $$\begin{array}{c} E{\left\|\nabla l_{k}\left(\theta ;x\right)\right\|}^{2}\leq \sigma^{2}k\in K,\sigma >0\text{,}\\ s.t.\nabla f_{k}\left(\theta^{t,e}\right)=\nabla l_{k}\left(\theta^{t,e};x\right)e\in I,t\in T,x\in D\text{.} \end{array}$$

**Theorem 1 .**

Suppose Assumptions 1 and 2 hold, when the step size is set as $\eta =\left(1/L\sqrt{T}\right)$, the convergence of FedAwo with nonconvex loss functions satisfies:

(12) $$\begin{array}{c} \frac{1}{T}\overset{T}{\underset{t=1}{\sum }}E{\left\|\nabla f\left(\theta^{t}\right)\right\|}^{2}\leq \frac{2L}{I\sqrt{T}}\left[f\left(\theta^{0}\right)-f\left(\theta^{*}\right)\right]+\frac{I^{2}\sigma^{2}K}{L^{2}T}\overset{K}{\underset{k=1}{\sum }}{\left(q_{k}^{t}\right)}^{2}+\frac{I\sigma^{2}K}{\sqrt{T}}\overset{K}{\underset{k=1}{\sum }}{\left(q_{k}^{t}\right)}^{2}, \end{array}$$

where *f*(*θ*^*∗*^) denotes the minimum value of (1), and *f*(*θ*^0^) denotes the initialized value of (1).

**Proof**

In order to prove inequality (11) is true, *E*‖∇*f*(*θ*^*t*^)‖ would be deduced first.

According to (5), *θ*^*t*^ can be defined as 2.1 ng, and the amount of data can be expressed as follows (Appendix A):

(13) $$\begin{array}{c} \theta^{t+1}=\overset{K}{\underset{k=1}{\sum }}q_{k}\cdot \theta_{k}^{t}=\theta^{t}-\eta \overset{K}{\underset{k=1}{\sum }}\left(q_{k}\cdot \overset{I-1}{\underset{e=0}{\sum }}\nabla f_{k}\left(\theta^{t,e}\right)\right)\text{.} \end{array}$$

Since *f*(.)is *L* − smooth, *E*[*f*(*θ*^*t*+1^)], it can be derived as follows:

(14) $$\begin{array}{c} E\left[f\left(\theta^{t+1}\right)\right]\leq E\left[f\left(\theta^{t}\right)\right]+E\left[\langle \nabla f\left(\theta^{t+1}\right),\theta^{t+1}-\theta^{t}\rangle +\frac{L}{2}E\left[{\left\|\theta^{t+1}-\theta^{t}\right\|}^{2}\right]\right]\\ =E\left[f\left(\theta^{t}\right)\right]+\frac{L}{2}E\left[{\left\|\theta^{t+1}-\theta^{t}\right\|}^{2}\right]\\ -\eta E\left[\langle \nabla f\left(\theta^{t}\right),\overset{K}{\underset{k=1}{\sum }}\left(q_{k}^{t}\cdot \overset{I-1}{\underset{e=0}{\sum }}\nabla f_{k}\left(\theta^{t,e}\right)\right)\rangle \right]\text{.} \end{array}$$

The expectation of the inner product in inequality (14) can be derived as inequality (15) (Appendix B):

(15) $$\begin{array}{c} E\left[\langle \nabla f\left(\theta^{t}\right),\theta^{t+1}-\theta^{t}\rangle \right]=-\eta E\left[\langle \left(\nabla f\left(\theta^{t}\right),\overset{K}{\underset{k=1}{\sum }}\left(q_{k}^{t}\cdot \overset{I-1}{\underset{e=0}{\sum }}\nabla f\left(\theta^{t,e}\right)\right)\right.\rangle \right]\\ \leq -\frac{\eta I}{2}E\left[{\left\|\nabla f\left(\theta^{t}\right)\right\|}^{2}\right]+\frac{1}{2}\eta^{3}I^{3}\sigma^{2}L^{2}K\overset{K}{\underset{k=1}{\sum }}{\left(q_{k}^{t}\right)}^{2}\text{.} \end{array}$$

According to inequality ‖∑_*k*=1_^*K*^*z*_*k*_‖^2^ ≤ *K*∑_*k*=1_^*K*^‖*z*_*k*_‖^2^, *E*[‖*θ*^*t*+1^ − *θ*^*t*^‖^2^] of inequality (14) can be rewritten as (16) (Appendix C):

(16) $$\begin{array}{c} E\left[{\left\|\theta^{t+1}-\theta^{t}\right\|}^{2}\right]=E\left[{\left\|\theta^{t}-\eta \nabla f\left(\theta^{t}\right)-\theta^{t}\right\|}^{2}\right]\\ \left.\leq \eta^{2}K\overset{K}{\underset{k=1}{\sum }}{\left(q_{k}^{t}\right)}^{2}E\left[{\left\|\overset{I-1}{\underset{e=0}{\sum }}\nabla f_{k}\left(\theta^{t,e}\right)\right\|}^{2}\right]\right]\\ \leq \eta^{2}I^{2}\sigma^{2}K\overset{K}{\underset{k=1}{\sum }}{\left(q_{k}^{t}\right)}^{2}\text{.} \end{array}$$

Substituting inequality (15) and (16) into inequality (14) yields inequality:

(17) $$\begin{array}{c} E\left[f\left(\theta^{t+1}\right)\right]\leq E\left[f\left(\theta^{t+1}\right)\right]+\frac{L}{2}E\left[{\left\|\theta^{t+1}-\theta^{t}\right\|}^{2}\right]\\ +E\left[\langle \nabla f\left(\theta^{t}\right),\theta^{t+1}-\theta^{t}\rangle \right]\\ \leq E\left[f\left(\theta^{t}\right)\right]-\frac{\eta I}{2}E\left[{\left\|\nabla f\left(\theta^{t}\right)\right\|}^{2}\right]\\ +\frac{1}{2}\eta^{3}I^{3}L^{2}\sigma^{2}K\overset{K}{\underset{k=1}{\sum }}{\left(q_{k}^{t}\right)}^{2}+\frac{1}{2}\eta^{2}I^{2}L\sigma^{2}K\overset{K}{\underset{k=1}{\sum }}{\left(q_{k}^{t}\right)}^{2}\text{.} \end{array}$$

Dividing (17) both sides by (*ηI*/2) and rearranging terms yield inequality:

(18) $$\begin{array}{c} E\left[{\left\|\nabla f\left(\theta^{t}\right)\right\|}^{2}\right]\leq \frac{2}{\eta I}\left[E\left[f\left(\theta^{t}\right)\right]+\frac{1}{2}\eta^{3}I^{3}L^{2}\sigma^{2}K\overset{K}{\underset{k=1}{\sum }}{\left(q_{k}^{t}\right)}^{2}+\frac{1}{2}\eta^{2}I^{2}L\sigma^{2}K\overset{K}{\underset{k=1}{\sum }}{\left(q_{k}^{t}\right)}^{2}-E\left[f\left(\theta^{t+1}\right)\right]\right]\text{.} \end{array}$$

Summing over *t* ∈ {0,1,…, *T* − 1} and dividing both sides by *T* yield inequality:

(19) $$\begin{array}{c} \frac{1}{T}\overset{T-1}{\underset{t=0}{\sum }}E{\left\|\nabla f\left(\theta^{t}\right)\right\|}^{2}\frac{2}{\eta IT}\leq \overset{T-1}{\underset{t=0}{\sum }}\left[E\left[f\left(\theta^{t}\right)\right]+\frac{1}{2}\eta^{3}I^{3}L^{2}\sigma^{2}K\overset{K}{\underset{k=1}{\sum }}{\left(q_{k}^{t}\right)}^{2}+\frac{1}{2}L\eta^{2}I^{2}\sigma^{2}K\overset{K}{\underset{k=1}{\sum }}{\left(q_{k}^{t}\right)}^{2}-E\left[f\left(\theta^{t+1}\right)\right]\right]\\ \leq \frac{2}{\eta IT}\left[f\left(\theta^{0}\right)-f\left(\theta^{*}\right)\right]+\eta^{2}I^{2}L\sigma^{2}K\overset{K}{\underset{k=1}{\sum }}{\left(q_{k}^{t}\right)}^{2}+L\eta I\sigma^{2}K\overset{K}{\underset{k=1}{\sum }}{\left(q_{k}^{t}\right)}^{2}\text{.} \end{array}$$

Finally, substituting $=\left(1/L\sqrt{T}\right)$ into (18) yields the desired result (12). So Theorem 1 is true.

### 3.2. Strongly Convex Loss Functions

Compared with nonconvex loss functions, the convergence analysis of convex loss functions usually adds Assumption 3 [13, 16, 22].

**Assumption 3 .**

The loss functions *f*(.), *f*_*k*_(.), *l*(.) are *μ* strongly convex, and for any *α*, *β* ∈ *R*^*d*^ and any *x* ∈ *D*, there is inequality.

(20) $$\begin{array}{c} \langle \nabla l\left(\alpha ;x\right)-\nabla l\left(\beta ;x\right),\alpha -\beta \rangle \geq \mu {\left\|\alpha -\beta \right\|}^{2}\text{.} \end{array}$$

**Theorem 2 .**

Suppose Assumptions 1to 3 hold, for any *t* > *t*_0_ (*t*_0_ is a constant), when the step size is set as *η*_*t*_=(1/*μ* · *t*), the convergence of FedAwo with strongly convex loss functions satisfies:

(21) $$\begin{array}{c} E{\left\|\theta^{t}-\theta^{*}\right\|}^{2}\leq \frac{t_{0}}{t}E{\left\|\theta^{t_{0}}-\theta^{*}\right\|}^{2}+\frac{B}{t}\text{,} \end{array}$$

where $B\overset{de\;f}{=}\left(I^{2}\sigma^{2}K/\mu^{2}\right)\sum_{k=1}^{K}{\left(q_{k}^{t}\right)}^{2}$.

**Proof**

(22) $$\begin{array}{c} E{\left\|\theta^{t}-\theta^{*}\right\|}^{2}=E\left[{\left\|\theta^{t}-\eta_{t}\nabla f\left(\theta^{t}\right)-\theta^{*}\right\|}^{2}\right]\leq \left(1-2\mu \eta_{t}\right)E\left[{\left\|\theta^{t}-\theta^{*}\right\|}^{2}\right]+\eta_{t}^{2}I^{2}\sigma^{2}K\overset{K}{\underset{k=1}{\sum }}{\left(q_{k}^{t}\right)}^{2}\text{.} \end{array}$$

Substituting *η*_*t*_=(1/*μ* · *t*) into inequality (22) (Appendix D) yields:

(23) $$\begin{array}{c} E{\left\|\theta^{t+1}-\theta^{*}\right\|}^{2}\leq \left(1-\frac{2}{t}\right)E\left[{\left\|\theta^{t}-\theta^{*}\right\|}^{2}\right]+\frac{I^{2}\sigma^{2}K}{\mu^{2}t^{2}}\overset{K}{\underset{k=1}{\sum }}{\left(q_{k}^{t}\right)}^{2}\text{.} \end{array}$$

For the sake of simplicity, set $\lambda^{t}\overset{de\;f}{=}E\left[{\left\|\theta^{t}-\theta^{*}\right\|}^{2}\right]$, $B\overset{de\;f}{=}\left(I^{2}\sigma^{2}K/\mu^{2}\right)\sum_{k=1}^{K}{\left(q_{k}^{t}\right)}^{2}$. The inequality (23) is rewritten as inequality:

(24) $$\begin{array}{c} \lambda^{t+1}\leq \left(1-\frac{2}{t}\right)\lambda^{t}+\frac{B}{t^{2}}\text{.} \end{array}$$

Next, the induction would be used to derive Theorem 2. Obviously, inequality (11) holds for *t*=*t*_0_, and then, assuming that inequality (21) is true when *s* > *t*_0_. Then, we have

(25) $$\begin{array}{c} \lambda^{t}\leq \frac{t_{0}}{s}\lambda^{t_{0}}+\frac{B}{s}\text{.} \end{array}$$

Next, from inequality (24) and (25), we obtain inequality as follows:

(26) $$\begin{array}{c} \lambda^{t}\leq \left(1-\frac{2}{s}\right)\left(\frac{t_{0}}{s}\lambda^{t_{0}}+\frac{B}{s}\right)+\frac{B}{s^{2}}\leq \frac{t_{0}}{s+1}\lambda^{t_{0}}+\frac{B}{s+1}\text{.} \end{array}$$

Therefore, inequality (21) is true; i.e., Theorem 2 is true.

## 4. Federated Learning Enhancement Algorithm for Automatic Weight Optimization (FedAwo ^*∗*^)

System heterogeneity is caused by the client's computing power, storage capacity, load capacity, and network environment, and it means that the converged clients still need to carry out model training for the specified epoch. This phenomenon results in the computing resource waste and energy waste in clients. Therefore, we further optimize the FedAwo algorithm and propose an enhanced algorithm (FedAwo ^*∗*^). Based on the FedAwo algorithm, the FedAwo ^*∗*^ algorithm adds an adaptive training round optimization algorithm to the client, which can effectively reduce the model training overhead of clients.

The above phenomenon is common in federated learning, but traditional federated learning algorithms do not pay attention to this problem, and this phenomenon is aggravated with the progress of federated learning, which leads to a large number of invalid calculations in the client and adds a lot of meaningless computational overheads. Therefore, it is necessary to add discriminant conditions for model convergence in local training. This is where the FedAwo ^*∗*^ algorithm is optimized for the FedAwo algorithm. This method returns to the server a local model that satisfies the convergence criteria, even if the specified epoch has not been completed. This idea seems to be similar to that of the FedProx algorithm [2], but the starting points of them are completely different. FedProx is to solve the problem of struggling, while FedAwo ^*∗*^ is to reduce training costs. When the model trained by the client reaches the convergence criteria we set, the local training would automatically stop even if the training numbers are less than the epoch set by the system. And local converged model would been returned to the server, so as to reduce the computational overhead of the clients.

The specific process of FedAwo ^*∗*^ is as follows:

1. In each epoch, clients save *l*^*e*^ of the current epoch and subtracts the previous round *l*^*e*−1^ to get the difference ∇*l*^*e*^=|*l*^*e*^ − *l*^*e*−1^|.
2. Judging the convergence of clients, if ∇*l*^*e*^ < *ε* and ∇*l*^*e*+1^ < *ε*, |*δ* − *l*^*e*^| < *ε*, *θ*_*k*_^*t*^ is considered converged and would be returned to the server. *ε* represents a very small parameter and *δ* represents a parameter close to the global model convergence loss. The value is adjusted according to the specific situation. In section 6, we would set *δ*=0 and *ε*=0.001.
3. If the conditions in II cannot meet the specified criterion, after training the specified criterion, *θ*_*k*_^*t*^ would be returned to the server.

In Algorithm 2, we need to define the initial global model *θ*^0^, the initial adaptive learning model *ϑ*^0^, the initial weight value *q*_*k*_^0^, the initial loss function difference ∇*l*^0^=0, and other parameters *ϵ*, *δ*. The server broadcasts the global model *θ*^0^ to all clients within the specified time *T* of the system. The client uses local data to train the model for the specified epoch *I* and then returns the model *θ*_*k*_^*t*,*I*^ to the server. At the same time, in each local training epoch, we would record the difference ∇*l*^*e*^ between the loss function of this epoch and the previous epoch. When the difference ∇*l*^*e*^ between the loss functions of two consecutive epoch is very close, or the difference between these two epochs is less than *ϵ*, we consider that the local model has converged at this time and immediately return this model to the server. This process is shown in 2–14 of Algorithm 2, which represents the process of local model training. Then, in the server, the optimal weight value *q*_*k*_^*∗*^ is obtained through the adaptive learning model *ϑ*^0^. The model aggregation is carried out according to the optimal weight value *q*_*k*_^*∗*^, and the latest global model *θ*^*t*+1^ is obtained. This process is shown in 15–18 of Algorithm 2, which represents the process of model aggregation.

The algorithm reduces the computational overhead of clients by dynamically performing local-training epochs. According to a large number of experiments, we found that in the process of federated learning, some clients have converged before performing the number of specified epochs. Following the previous federated learning algorithm, these clients still need to perform training until the specified epoch. This process inevitably results in the waste of computing resources [2]. Therefore, the FedAwo ^*∗*^ algorithm adaptively judges whether the SGD process converges during the client training. If the convergence conditions are reached before the specific epoch, the SGD would be stopped and the converged local model would be returned to the server. Otherwise, the SGD would continue and stop after reaching the specified epoch.

## 5. Experiments

### 5.1. Experimental Environment

In order to analyze the performance of FedAwo and FedAwo ^*∗*^ algorithms, we established an experimental environment based on PyTorch 1.10.1 and CUDA 10.2. The software environment is Python 3.8. The hardware environment is 3.60 GHz AMD Ryzen 7 3700X 8core processor CPU, 16.00 GB, Win10 64 bit, and NVIDIA GeForce RTX 2070 system. The simulation experiment strictly follows the protocols and rules that may be used in distributed federated learning [35]. More details of the experimental environment are shown in Table 3.

### 5.2. Experimental Setup

In this paper, MNIST and Fashion-MNIST datasets are selected as experimental datasets to verify the performance and stability of FedAwo and FedAwo ^*∗*^ algorithms. MNIST and Fashion-MNIST are two image datasets. In the experiment, we normalize the two datasets, respectively. For IID dataset partition, data samples are evenly and randomly distributed to clients. For nonIID dataset partitions, data samples are sorted by their labels and divided into 2K groups, and each client receives two groups (i.e., samples corresponding to two labels).

For MNIST, the dataset has 60000 training samples and 10000 test samples. It is an image dataset containing 0–9 hand-written digits, and each sample contains 28 × 28 pixels. We set a total of *K* = 100 clients, and we allocate 600 training samples for each client. In addition, when using the FedAwo algorithm, we get 2000 data from 10000 test datasets and take these data as the server dataset *D*_*s*_ for adaptive learning of weight distribution and the remaining 8000 data as test datasets. For comparison, we configured the same CNN model according to the method proposed in [1]. The model has two 5 × 5 convolution layers of CNN (the first has 32 channels, the second has 64 channels, and each channel is followed by 2 × 2 maximum pool), and one has 512 units, ReLU activation, and final Softmax output layer.

For Fashion-MNIST, the dataset also has 60000 training samples and 10000 test samples. It is an image dataset containing different commodities, and each sample also contains 28 × 28 pixels. Other experimental settings are consistent with the MNIST dataset.

The specific experimental setup details are as follows: we set the learning rate to 0.01, batch size to 64, and epoch to 5. Since the MNIST and Fashion-MNIST datasets have the same input and output and are both image datasets, we set the same CNN model. The details of the specific model settings are shown in Table 4.

### 5.3. Results of the Experiment

We chose the most classic and widely used FedAvg, FedProx, and FedAdp algorithms as the baselines of the experiments.

**Figure 1:**
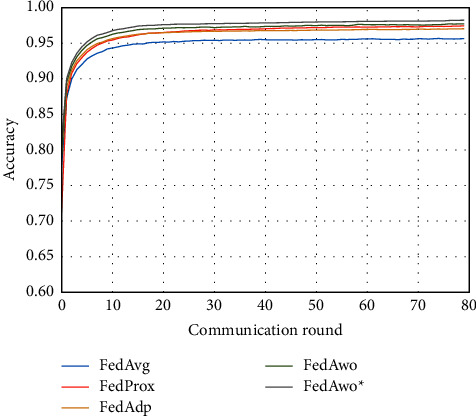
Global model accuracy (IID).

**Figure 2:**
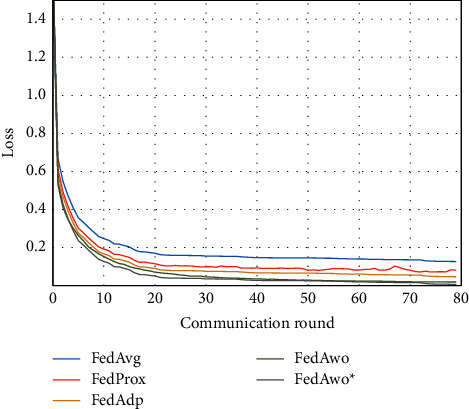
Global model loss (IID).

For the MNIST dataset, we first used the data distribution of IID to compare FedAwo, FedAwo ^*∗*^, FedAvg, FedProx, and FedAdp algorithms. As shown in Figures 1 and 2, we could see that under the dataset with IID distributed data, the five algorithms converge in 10–15 communication rounds. FedAvg has slower convergence speed and lower accuracy of the global model than the other four algorithms, but it is not clear.

**Figure 3:**
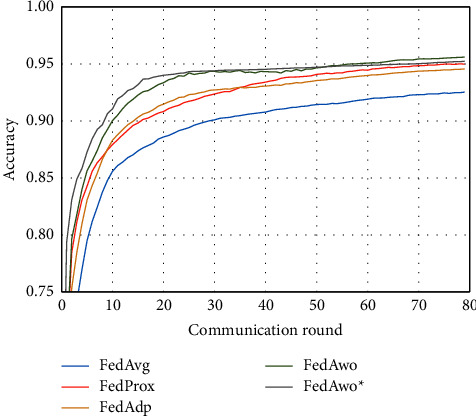
Global model accuracy (statistical heterogeneity).

**Figure 4:**
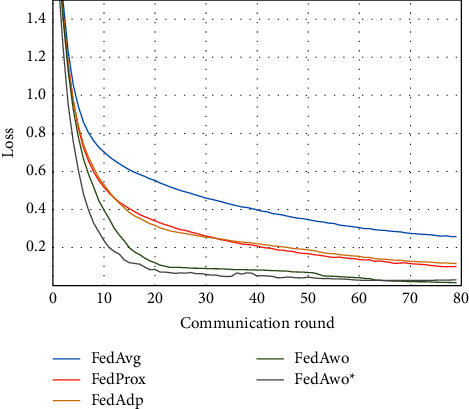
Global model loss (statistical heterogeneity).

NonIID experiments heavily distribute skewed data to individual clients, and the results of the experiment are shown in Figures 3 and 4. In Figures 1 and 2, we could see that the convergence rates of the five algorithms were affected by statistical heterogeneity. The FedAvg algorithm was seriously affected, which led to a significant decrease in the convergence speed, and converged after the 70th communication round. At the same time, the quality of the global model was obviously inferior to the global model under the IID condition. Due to the addition of the near term to the loss function, the quality of the global model was not affected in the FedProx algorithm, but the convergence speed was still slowed down. The same is true for the FedAdp algorithm. For FedAwo and FedAwo ^*∗*^ algorithms, both the convergence speed and the quality of the global model were minimally affected by statistical heterogeneity, and they reached convergence around the 30th communication round.

**Figure 5:**
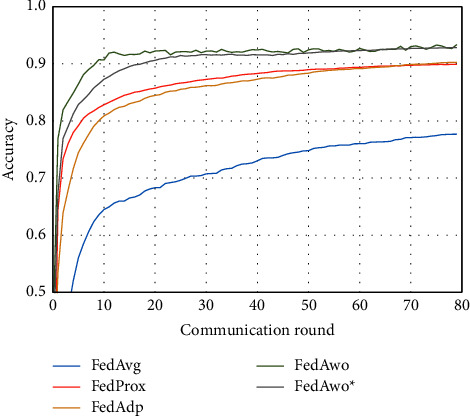
Global model accuracy (statistical heterogeneity and system heterogeneity).

**Figure 6:**
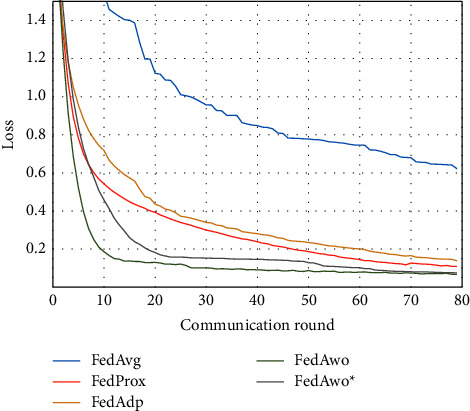
Global model loss (statistical heterogeneity and system heterogeneity).

We also simulated both systematic and statistical heterogeneity of federated learning. Obviously, the influence of heterogeneity on the global model was further increased. It could be seen from Figures 5 and 6 that the FedAvg algorithm had a great impact on the convergence speed and global model quality. The model did not completely converge until round 80. The convergence speed of FedProx and FedAdp was not significantly slowed down compared with the condition of only statistical heterogeneity, but the quality of the global model was degraded. For FedAwo, both the convergence rate and the quality of the global model were still minimally affected, while FedAwo ^*∗*^ had some fluctuations under the influence of system heterogeneity. The convergence speed and global model quality of FedAwo and FedAwo ^*∗*^ algorithms were better than those of FedAvg, FedProx, and FedApd baseline algorithms.

In order to ensure the superiority of FedAwo and FedAwo ^*∗*^ algorithms, we conducted the Friedman test on the model accuracy and loss of these five algorithms and obtained the results of stat = 14.68, *p* value = 0.00184 and stats = 10.24, *p* value = 0.02626. The Friedman test can only show that there are differences between the accuracy and loss of the models, but it cannot show which model is better. Therefore, we conducted the Nemenyi test on the above algorithms to further verify whether there is a significant difference between the two models. According to the results shown in Table 5, it can be concluded that FedAwo and FedAwo ^*∗*^ algorithms are superior to the other three algorithms.

In addition to accuracy and loss, we also cited the final precision, recall, AUC, and F1 values of the global model as performance indicators to compare the five algorithms under statistical heterogeneity, as shown in Table 6.

**Figure 7:**
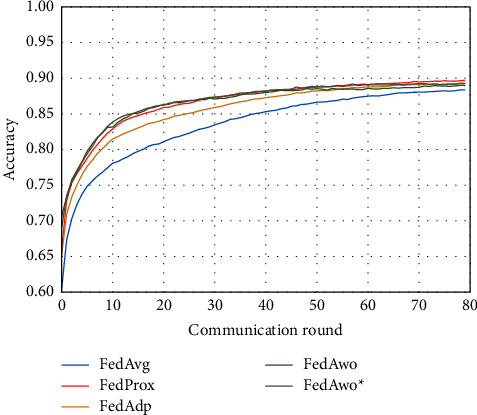
Global model accuracy (IID).

**Figure 8:**
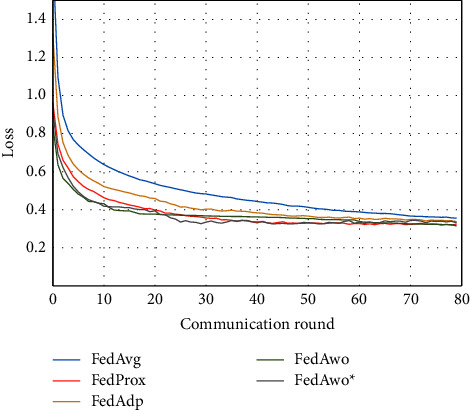
Global model loss (IID).

For the Fashion-MNIST dataset, we obtained similar conclusions as in the MNIST dataset. According to Figures 7 and 8, the convergence speed of the FedAvg algorithm would be slower under the IID condition, and the other four algorithms were not much different.

**Figure 9:**
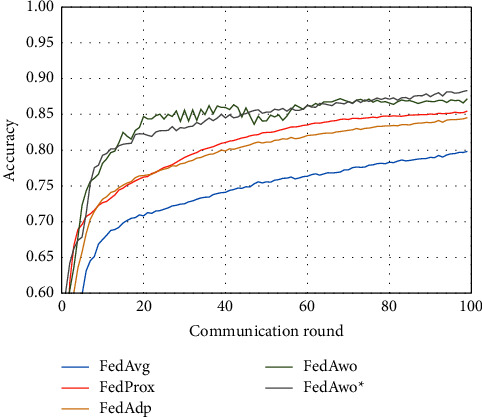
Global model accuracy (statistical heterogeneity).

**Figure 10:**
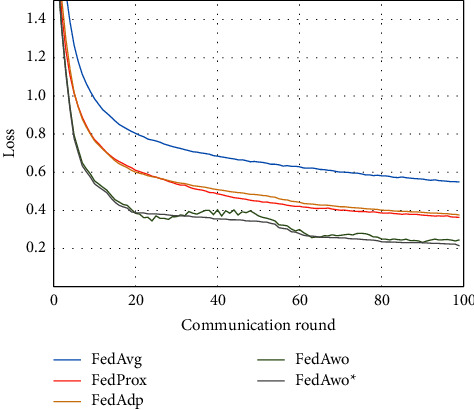
Global model loss (statistical heterogeneity).

According to Figures 9 and 10, the experimental results were also similar to those in the MNIST dataset of the influence with only statistical heterogeneity.

**Figure 11:**
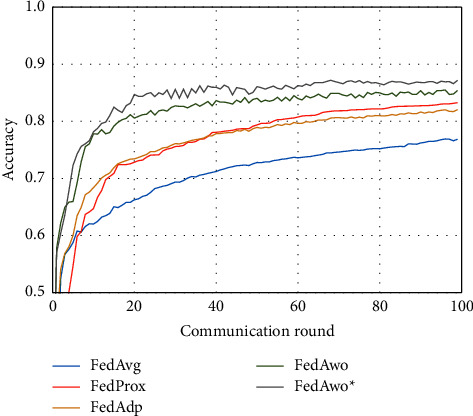
Global model accuracy (statistical heterogeneity and system heterogeneity).

**Figure 12:**
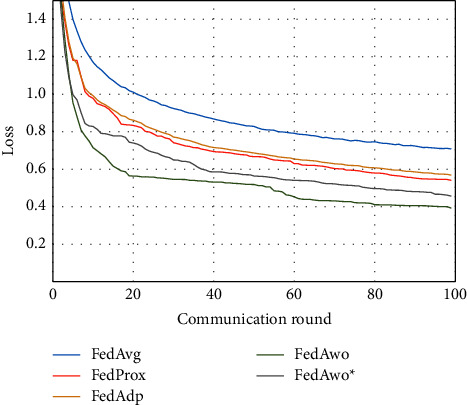
Global model loss (statistical heterogeneity and system heterogeneity).

In Figures 9–12, we can see that although the FedAwo and FedAwo ^*∗*^ algorithms have some fluctuations, their convergence speed and model accuracy are better than those of the baseline algorithms.

For the Fashion-MNIST dataset, we conducted the Friedman test on the model accuracy and loss of the five algorithms, and the results obtained were stat = 13.27, *p* value = 0.00181 and stats = 10.24, *p* value = 0.02626. We conducted the Nemenyi test on the above algorithms to further verify whether there is a significant difference between the two models. According to the results in Table 7, it can be concluded that FedAwo and FedAwo ^*∗*^ algorithms are superior to the other three algorithms.

Similarly, for the Fashion-MNIST dataset, we also cited the final precision, recall, AUC, and F1 values of the global model as performance indicators to compare the five algorithms under statistical heterogeneity, as shown in Table 8.

**Figure 13:**
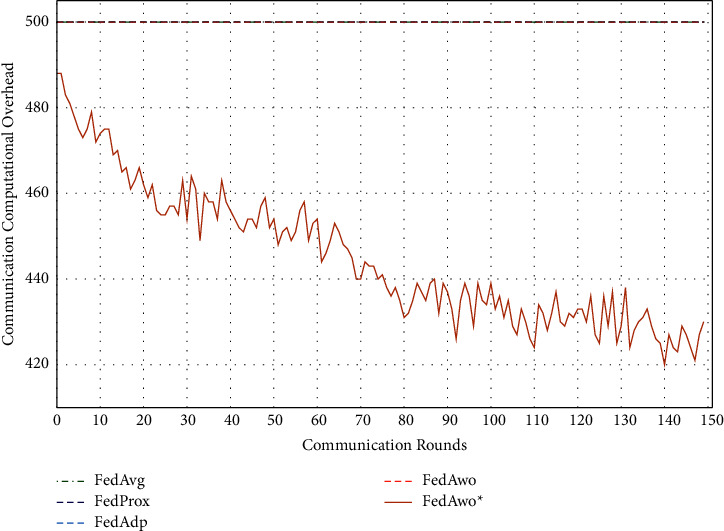
Computational overhead (IID).

**Figure 14:**
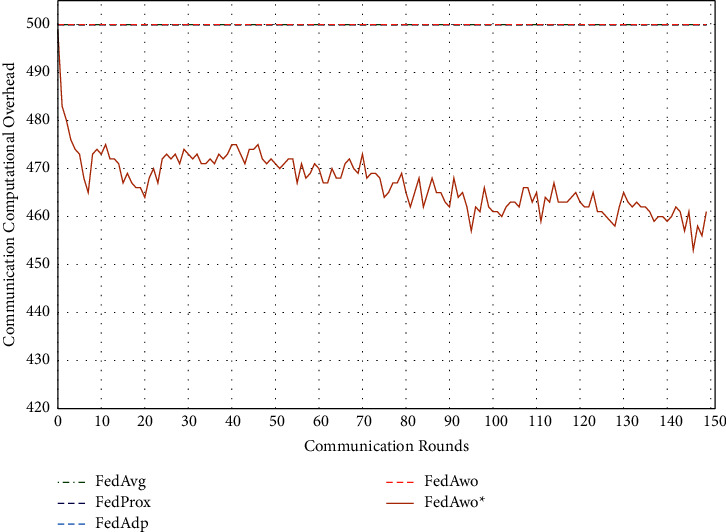
Computational overhead (statistical heterogeneity).

In addition, we tested the computational overhead of four algorithms under the IID condition and nonIID condition. The results of IID condition are shown in Figures 13 and 14, and the other four algorithms did not judge whether the local model converged, so clients would train 5 epochs in each round. The total calculation amount of 100 clients in a communication round is 500. Due to the IID dataset, the convergence speed of the local model and the global model was fast. When the model and global model was close to convergence, clients would save more computing resources.

**Figure 15:**
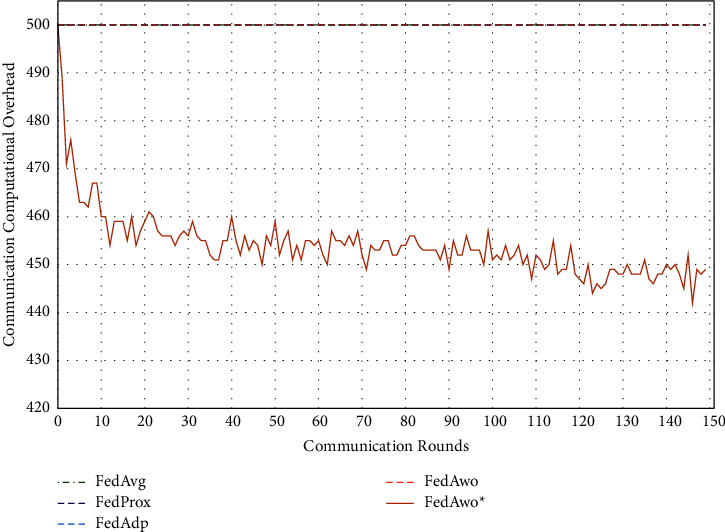
Computational overhead (IID).

**Figure 16:**
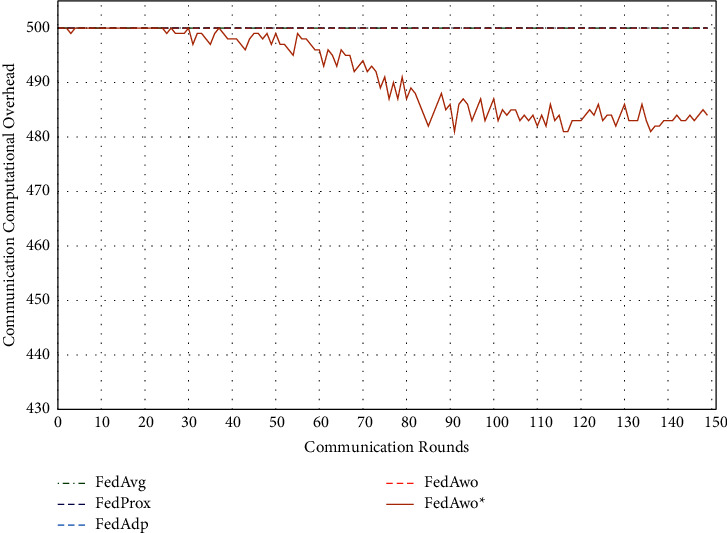
Computational overhead (statistical heterogeneity).

Under the condition of nonIID (statistical heterogeneity), FedAwo ^*∗*^ can still save computing resources of clients. However, compared with the IID condition, the convergence speed was slower in the case of statistical heterogeneity, and the saving effect of saving computing resources in the FedAwo ^*∗*^ algorithm was slightly worse, which is shown in Figures 13–16.

### 5.4. Discussion on Experiment

According to Figures 1 and 2, in the MNIST dataset, we can see that each federated learning algorithm has similar performance without heterogeneity. When we use the local dataset with statistical heterogeneity, as shown in Figures 3 and 4, the global model accuracy and convergence speed of the FedAvg algorithm are significantly reduced. The global model accuracy of the FedProx algorithm and the FedAdp algorithm is not affected, but the convergence speed is significantly reduced, reaching convergence in the 70th round. However, the global model accuracy and the convergence speed of the FedAwo algorithm and the FedAwo ^ ^*∗*^^ algorithm are almost not affected by statistical heterogeneity and can reach convergence 20 rounds before. On this basis, we add system heterogeneity. When two types of heterogeneity exist at the same time, heterogeneity has a more significant negative impact on model aggregation. As shown in Figures 5 and 6, the global accuracy and convergence speed of the FedAvg, FedProx, and FedAdp algorithms are significantly reduced. The FedAwo and FedAwo ^*∗*^ algorithms have received a slight impact, but the global model accuracy can still reach 90% and can converge within 20 rounds. In the Fashion-MNIST dataset, we get consistent results, as shown in Figures 7–12. Through the above experiments, it fully reflects the optimal weight value calculated according to the adaptive learning algorithm; compared with the weight value assigned by the traditional federated learning algorithm according to the amount of client data, it has significant advantages. The FedAwo ^*∗*^ algorithm optimizes the computational cost of the FedAwo algorithm for the client. As shown in Figures 13–16, FedAwo ^*∗*^ can significantly reduce the computing overhead of the client and is applicable to the situation of both IID and heterogeneity.

Through the above experiments, we can clearly find that the ability of FedAwo and FedAwo ^*∗*^ algorithms to solve the heterogeneity of federated learning is better than that of the other three baseline algorithms. Even under the condition of system heterogeneity and statistical heterogeneity, the algorithm in this paper can still converge quickly and ensure excellent global model quality. In addition, the algorithm in this paper is still applicable to IID. Therefore, FedAwo and FedAwo ^*∗*^ algorithms are universal, and they can be applied to most federal learning scenarios. The FedAwo ^*∗*^ algorithm optimizes the convergence criterion of the local model. As shown in Figures 13 and 16, FedAwo ^*∗*^ significantly saves the computing overhead of the client compared with other algorithms. Therefore, FedAwo ^*∗*^ is an adaptive weight optimization federated learning algorithm that can effectively solve the heterogeneity and save the computational overhead. Compared with existing algorithms, it has great advantages.

## 6. Conclusion

We investigate an automatic local model weight optimization strategy to reduce the negative effects of systematic and statistical heterogeneity in federated learning and propose federated learning algorithms FedAwo and FedAwo ^*∗*^. The FedAwo algorithm can improve the convergence speed of the global model and obtain a global model with higher accuracy, and the enhancement algorithm FedAwo ^*∗*^ can reduce the training overhead. Experimental results verify the superiority of our proposed schemes in terms of convergence speed and global model accuracy, as well as the effectiveness of FedAwo ^*∗*^ in saving the client-computing overhead. In this paper, we combine adaptive learning with federated learning to solve the heterogeneity problem and have achieved remarkable results. This paper puts forward a new idea to solve the negative impact of heterogeneity in federated learning.

## 7. Future Work

However, the FedAwo and FedAwo ^*∗*^ algorithms also have some instability. As shown in Figures 9 and 10, in the Experiment section, the global model shows the zig-zag spike phenomenon when it is close to convergence. The reason for this phenomenon is that the learning rate is too high when the algorithm is about to converge fast. In the future work, we hope to improve the zig-zag spike phenomenon by dynamically adjusting the learning rate. In addition, we will further improve the adaptive learning model $\vartheta^0$ in future work to further improve the performance of the FedAwo algorithm.

## Figures and Tables

**Algorithm 1:**
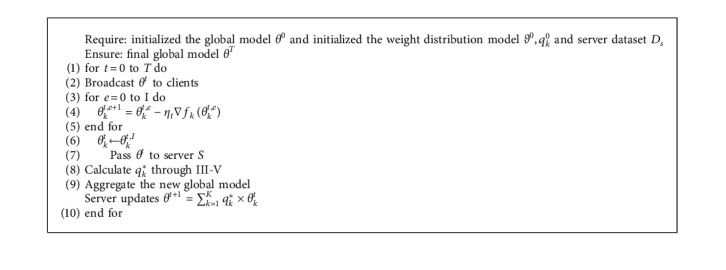
FedAwo (federated learning enhancement algorithm for automatic weight optimal allocation).

**Algorithm 2:**
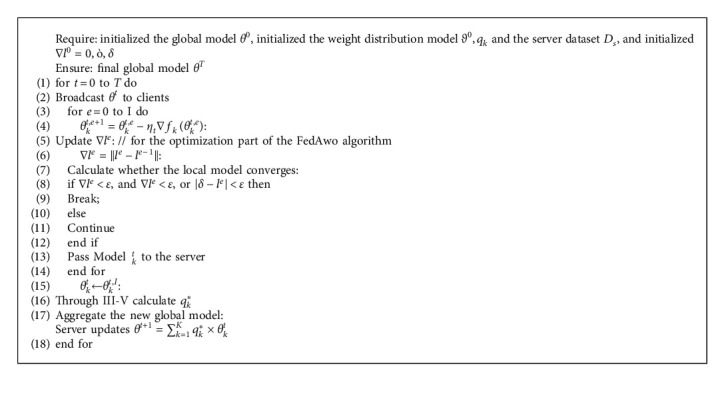
FedAwo ^*∗*^ (improved optimization algorithm of FedAwo).

**Table 1:** Limitations of the approach in federated learning.

Approach	Challenges of the approach
Modifying the loss function of clients [2, 10–12, 14]	The approach increases the computational overhead of the client
Approach of sampling [4, 15–17]	The approach has low accuracy of the final global model
Heterogeneous quantization [18]	The actual quantification standard is not specific
Approach of local gradient tracking [19, 20]	The approach ignored the statistical heterogeneity
Approach of combining adaptive learning [13, 21, 22]	The previous work was not intended to solve heterogeneity

**Table 2:** Adjustment of the loss function.

Existing loss function	Updated loss function
*θ* ^*t*+1^=∑_*k*=1_^*K*^*p*_*k*_ · *θ*_*k*_^*t*^	*θ* ^*t*+1^=∑_*k*=1_^*K*^*q*_*k*_ · *θ*_*k*_^*t*^

**Table 3:** Details of the experimental environment.

Environment detail specifications	
Operating system	Microsoft Windows 10
Processor	AMD Ryzen 7 3700X 8-core
Architecture	64-Bit
Memory allotted	4 GB
GPU	NVIDIA GeForce RTX 2070
Language	Python
Framework	PyTorch, FastAI
Libraries used	pandas, NumPy, Matplotlib, argparse

**Table 4:** Layered architecture of the experimental model.

Layers	Kernel size	Parameters	Tensor size
Convolution	5 × 5 (conv)	Stride = 1	1 × 32
Pooling	2 × 2 (ma × pool)	Stride = 2	—
Convolution	5 × 5 (conv)	Stride = 1	32 × 64
Pooling	2 × 2 (ma × pool)	Stride = 2	—
Linear	1 × 1	—	7 × 7 × 64 × 512
Linear	1 × 1	—	512 × 10

**Table 5:** Results of the Nemenyi test.

	FedAvg	FedAdp	FedProx	FedAwo	FedAwo
FedAvg	1.00	0.90	0.72	0.12	0.02
FedAdp	0.90	1.00	0.89	0.22	0.04
FedProx	0.72	0.89	1.00	0.72	0.32
FedAwo	0.12	0.22	0.72	1.00	0.90
FedAwo ^*∗*^	0.02	0.04	0.32	0.90	1.00

**Table 6:** Comparison of indicators between FedAwo and FedAwo ^*∗*^ with the state-of-art algorithms (MNIST).

	Precision (%)	Recall (%)	AUC	F1
FedAvg	93.8	93.2	0.975	0.944
FedProx	97.1	96.9	0.995	0.961
FedAdp	96.8	95.4	0.982	0.966
FedAwo	97.3	96.8	0.994	0.971
FedAwo ^*∗*^	96.9	96.9	0.995	0.967

**Table 7:** Results of the Nemenyi test.

	FedAvg	FedAdp	FedProx	FedAwo	FedAwo ^*∗*^
FedAvg	1.00	0.84	0.61	0.37	0.04
FedAdp	0.84	1.00	0.90	0.90	0.37
FedProx	0.61	0.90	1.00	0.90	0.61
FedAwo	0.37	0.90	0.90	1.00	0.84
FedAwo ^*∗*^	0.04	0.37	0.61	0.84	1.00

**Table 8:** Comparison of indicators between FedAwo and FedAwo ^*∗*^ with the state-of-art algorithms (Fashion-MNIST).

	Precision (%)	Recall (%)	AUC	F1
FedAvg	81.8	81.2	0.832	0.824
FedProx	86.1	85.4	0.875	0.871
FedAdp	86.8	85.2	0.872	0.866
FedAwo	87.3	86.2	0.896	0.861
FedAwo ^*∗*^	86.3	86.0	0.891	0.877

## Data Availability

The MNIST and Fashion-MNIST datasets used to support the findings of this study have been deposited in the (“https://www.kaggle.com/datasets/oddrationale/mnist-in-csv”) (“https://www.kaggle.com/datasets/zalando-research/fashionmnist”) repository ((DOI or OTHER PERSISTENT IDENTIFIER)). The MNIST and Fashion-MNIST datasets used to support the findings of this study are included within the article. The MNIST and Fashion-MNIST datasets used to support the findings of this study are included within the supplementary information file(s). The experimental code has been open source to the “https://github.com/amazing-yx/FedAwo.” Fashion-MNIST is available at https://www.kaggle.com/datasets/zalando-research/fashionmnist MNIST is available at https://www.kaggle.com/datasets/oddrationale/mnist-in-csv. Our experimental code for the manuscript is as follows: https://github.com/amazing-yx/FedAwo.

## Appendix

### A Proof of equation (13)

We expand equation (5) according to the SGD,

(A1) $$\begin{array}{c} \theta^{t+1}=\overset{K}{\underset{k=1}{\sum }}q_{k}\cdot \theta_{k}^{t}\\ =\theta^{t}-\eta \nabla f\left(\theta^{t}\right)\\ =\theta^{t}-\eta \overset{K}{\underset{k=1}{\sum }}\left(q_{k}\cdot \nabla f_{k}\left(\theta^{t}\right)\right)\\ =\theta^{t}-\eta \overset{K}{\underset{k=1}{\sum }}\left(q_{k}\cdot \overset{I-1}{\underset{e=0}{\sum }}\nabla f_{k}\left(\theta^{t,e}\right)\right)\text{.} \end{array}$$

### B Proof of inequality (15)

We expand the right half of equation (14)

(B1) $$\begin{array}{c} E\left[\langle \nabla f\left(\theta^{t}\right),\theta^{t+1}-\theta^{t}\rangle \right]=-\eta E\left[\langle \left(\nabla f\left(\theta^{t}\right),\overset{K}{\underset{k=1}{\sum }}\left(q_{k}^{t}\cdot \overset{I-1}{\underset{e=0}{\sum }}\nabla f\left(\theta^{t,e}\right)\right)\right.\rangle \right]\\ =-\eta \overset{I-1}{\underset{e=0}{\sum }}E\left[\langle \nabla f\left(\theta^{t}\right),\overset{K}{\underset{k=1}{\sum }}\left(q_{k}^{t}\cdot \nabla f\left(\theta^{t,e}\right)\right)\rangle \right]\\ =-\frac{\eta }{2}\overset{I-1}{\underset{e=0}{\sum }}E\left[\begin{array}{c} \left.{\left\|\nabla f\left(\theta^{t}\right)\right\|}^{2}+{\left\|\overset{K}{\underset{k=1}{\sum }}\left(q_{k}^{t}\cdot \nabla f_{k}\left(\theta^{t,e}\right)\right)\right\|}^{2}\right]\\ -{\left\|\nabla f\left(\theta^{t}\right)-\overset{K}{\underset{k=1}{\sum }}\left(q_{k}^{t}\cdot \nabla f_{k}\left(\theta^{t,e}\right)\right)\right\|}^{2} \end{array}\right]\\ \leq -\frac{\eta }{2}\overset{I-1}{\underset{e=0}{\sum }}E\left[{\left\|\nabla f\left(\theta^{t}\right)\right\|}^{2}-{\left\|\nabla f\left(\theta^{t}\right)-\overset{K}{\underset{k=1}{\sum }}\left(q_{k}^{t}\cdot \nabla f_{k}\left(\theta^{t,e}\right)\right)\right\|}^{2}\right]\\ \leq -\frac{\eta }{2}\overset{I-1}{\underset{e=0}{\sum }}E\left[{\left\|\nabla f\left(\theta^{t}\right)\right\|}^{2}-{\left\|\overset{K}{\underset{k=1}{\sum }}q_{k}^{t}\nabla f_{k}\left(\theta^{t}\right)-\overset{K}{\underset{k=1}{\sum }}q_{k}^{t}\nabla f_{k}\left(\theta^{t,e}\right)\right\|}^{2}\right]\\ \leq -\frac{\eta }{2}\overset{I-1}{\underset{e=0}{\sum }}E\left[E{\left\|\nabla f\left(\theta^{t}\right)\right\|}^{2}-K\overset{K}{\underset{k=1}{\sum }}{\left(q_{k}^{t}\right)}^{2}E{\left\|\nabla f\left(\theta^{t}\right)-\nabla f\left(\theta^{t,e}\right)\right\|}^{2}\right]\\ \leq -\frac{\eta }{2}\overset{I-1}{\underset{e=0}{\sum }}\left[E{\left\|\nabla f\left(\theta^{t}\right)\right\|}^{2}-KL^{2}\overset{K}{\underset{k=1}{\sum }}{\left(q_{k}^{t}\right)}^{2}E{\left\|\theta^{t}-\theta^{t,e}\right\|}^{2}\right]\\ \leq -\frac{\eta }{2}\overset{I-1}{\underset{e=0}{\sum }}\left[E{\left\|\nabla f\left(\theta^{t}\right)\right\|}^{2}-\eta^{2}KL^{2}\overset{K}{\underset{k=1}{\sum }}{\left(q_{k}^{t}\right)}^{2}E\left\|\overset{e-1}{\underset{j=0}{\sum }}\nabla f_{k}\left(\theta^{t,j}\right)\right\|\right]\\ \leq -\frac{\eta I}{2}E\left[{\left\|\nabla f\left(\theta^{t}\right)\right\|}^{2}\right]+\frac{1}{2}\eta^{3}I^{3}\sigma^{2}L^{2}K\overset{K}{\underset{k=1}{\sum }}{\left(q_{k}^{t}\right)}^{2}\text{.} \end{array}$$

### C Proof of inequality (16)

According to inequality ‖∑_*k*=1_^*K*^*z*_*k*_‖^2^ ≤ *K*∑_*k*=1_^*K*^‖*z*_*k*_‖^2^, *E*[‖*θ*^*t*+1^ − *θ*^*t*^‖^2^] of inequality (14) can be rewritten as (16)

(C1) $$\begin{array}{c} E\left[{\left\|\theta^{t+1}-\theta^{t}\right\|}^{2}\right]=E\left[{\left\|\theta^{t}-\eta \nabla f\left(\theta^{t}\right)-\theta^{t}\right\|}^{2}\right]\\ =E\left[{\left\|-\eta \nabla f\left(\theta^{t}\right)\right\|}^{2}\right]\\ =\eta^{2}E\left[{\left.\overset{K}{\underset{k=1}{\sum }}q_{k}^{t}\nabla f_{k}\left(\theta^{t}\right)\right\|}^{2}\right]\\ \left.=\eta^{2}E\left[{\left\|\overset{K}{\underset{k=1}{\sum }}q_{k}^{t}\overset{I-1}{\underset{e=0}{\sum }}\nabla f_{k}\left(\theta^{t,e}\right)\right\|}^{2}\right]\right]\\ \leq \eta^{2}K\overset{K}{\underset{k=1}{\sum }}{\left(q_{k}^{t}\right)}^{2}E\left[{\left\|\overset{I-1}{\underset{e=0}{\sum }}\nabla f_{k}\left(\theta^{t,e}\right)\right\|}^{2}\right]\\ \leq \eta^{2}I^{2}\sigma^{2}K\overset{K}{\underset{k=1}{\sum }}{\left(q_{k}^{t}\right)}^{2}\text{.} \end{array}$$

### D. Proof of inequality (22)

We take $B\overset{de\;f}{=}\left(I^{2}\sigma^{2}K/\mu^{2}\right)\sum_{k=1}^{K}{\left(q_{k}^{t}\right)}^{2}$ into inequality (21)

(D1) $$\begin{array}{c} E{\left\|\theta^{t}-\theta^{*}\right\|}^{2}=E\left[{\left\|\theta^{t}-\eta_{t}\nabla f\left(\theta^{t}\right)-\theta^{*}\right\|}^{2}\right]\\ =E\left[{\left\|\theta^{t}-\theta^{*}-\eta_{t}\overset{K}{\underset{k=1}{\sum }}q_{k}^{t}\nabla f_{k}\left(\theta^{t}\right)\right\|}^{2}\right]\\ =E\left[{\left\|\theta^{t}-\theta^{*}-\eta_{t}\overset{K}{\underset{k=1}{\sum }}q_{k}^{t}\overset{I-1}{\underset{e=0}{\sum }}\nabla f_{k}\left(\theta^{t,e}\right)\right\|}^{2}\right]\\ =E\left[{\left\|\theta^{t}-\theta^{*}\right\|}^{2}\right]-2E\left[\langle \theta^{t}-\theta^{*},\eta_{t}\overset{K}{\underset{k=1}{\sum }}q_{k}^{t}\overset{I-1}{\underset{e=0}{\sum }}\nabla f_{k}\left(\theta^{t,e}\right)\rangle \right]\\ +E\left[{\left\|\eta_{t}\overset{K}{\underset{k=1}{\sum }}q_{k}^{t}\overset{I-1}{\underset{e=0}{\sum }}\nabla f_{k}\left(\theta^{t,e}\right)\right\|}^{2}\right]\\ \leq E\left[{\left\|\theta^{t}-\theta^{*}\right\|}^{2}\right]-2\mu \eta_{t}E\left[{\left\|\theta^{t}-\theta^{*}\right\|}^{2}\right]\\ +E\left[{\left\|\eta_{t}\overset{K}{\underset{k=1}{\sum }}q_{k}^{t}\overset{I-1}{\underset{e=0}{\sum }}\nabla f_{k}\left(\theta^{t,e}\right)\right\|}^{2}\right]\\ \leq \left(1-2\mu \eta_{t}\right)E\left[{\left\|\theta^{t}-\theta^{*}\right\|}^{2}\right]+\eta_{t}^{2}I^{2}\sigma^{2}K\overset{K}{\underset{k=1}{\sum }}{\left(q_{k}^{t}\right)}^{2}\text{.} \end{array}$$
